# Exploring the Phytochemical, Pharmacological and Nutritional Properties of *Moringa oleifera*: A Comprehensive Review

**DOI:** 10.3390/nu16193423

**Published:** 2024-10-09

**Authors:** Surisetti Divya, Vinay Kumar Pandey, Ritik Dixit, Sarvesh Rustagi, Tejas Suthar, David Atuahene, Vivien Nagy, Diána Ungai, Abdelhakam Esmaeil Mohamed Ahmed, Béla Kovács, Ayaz Mukarram Shaikh

**Affiliations:** 1 Department of Pharmacology, Kanpur Institute of Technology and Pharmacy, Kanpur 208001, Uttar Pradesh, India; divya.pharmacy.50@gmail.com; 2 Research & Development Cell, Biotechnology Department, Manav Rachna International Institute of Research and Studies (Deemed to Be University), Faridabad 121004, Haryana, India; 3 Department of Pharmaceutical Chemistry, Advance Institute of Biotech & Paramedical Sciences, Kanpur 209217, Uttar Pradesh, India; ritikdixit045@gmail.com; 4 Department of Food Technology, School of Applied & Life Sciences, Uttaranchal University, Dehradun 248007, Uttarakhand, India; sarveshrustagi@gmail.com; 5 Independent Researcher, Chicago, IL 60616, USA; suthartejas2525@gmail.com; 6 Department of Veterinary Sciences, School of Agriculture and Veterinary Medicine, University of Turin, Grugliasco, I-10095 Turin, Italy; david.atuahene@unito.it; 7 Faculty of Agriculture, Food Science, and Environmental Management, Institute of Food Technology, University of Debrecen, Böszörményi út 138, 4032 Debrecen, Hungary; nagy.vivien@agr.unideb.hu; 8 Doctoral School of Nutrition and Food Sciences, University of Debrecen, Böszörményi út 138, 4032 Debrecen, Hungary; ungai@agr.unideb.hu (D.U.); ahmed.abdelhakam@agr.unideb.hu (A.E.M.A.); kovacsb@agr.unideb.hu (B.K.); 9 Faculty of Forestry, University of Khartoum, Khartoum North 13314, Sudan; 10 Faculty of Agriculture, Food Science and Environmental Management, Institute of Food Science, University of Debrecen, 4032 Debrecen, Hungary; 11 World Food Forum, I-00100 Rome, Italy

**Keywords:** *Moringa oleifera*, pharmaceutical properties, health benefits, nutrition, bioactive compounds

## Abstract

**Background:*** Moringa oleifera* is one of the most nutrient-packed species, commonly known as the drumstick tree or miracle tree, and has garnered substantial popularity for its health benefits, phytochemical profile, and therapeutic potential. *Moringa oleifera* is a plant that is native to the Indian subcontinent and has been used in traditional medicine for thousands of years owing to its nutritional and therapeutic properties. **Methods:** The leaves, seeds, pods, roots, and flowers of this plant are enriched with a wide range of bioactive compounds such as flavonoids, alkaloids, vitamins, minerals, and essential amino acids. Therefore, it is considered a reservoir crop for both nutritional and medicinal applications. **Result:** The recent rediscovery of the plant is at the forefront of changes in nutrition, medicine, and public health. Owing to its varied clinical applications, the plant is a potential candidate for research in new drug development and functional foods. **Conclusions:** Potential applications of *Moringa* compounds in the treatment of chronic diseases include antioxidant, anti-inflammatory, antimicrobial (bacterial or fungal), and anticancerous effects. In this review, various phytochemical extraction techniques, therapeutic properties, and applications are discussed.

## 1. Introduction

From the sub-Himalayan regions of India, Pakistan, Afghanistan, and Bangladesh, *Moringa oleifera* is the predominant pantropical species within the Monogeneric Moringaceae family. It is an all-purpose tree that is a lifeline for many communities [1]. It is commercially grown across several continents, regions, and countries including Africa, Hawaii, South and Central America, Mexico, and Asia. It is also known as the “horseradish tree” for its ground root taste, or the “drumstick tree” because its pods look like drumsticks when the seeds are not fully mature. Due to the oil made from the seeds of this plant, it has also been referred to as a “ben oil tree” [2]. In some areas, people consume the tender seed pods; fresh leaves are common ingredients in their food because they are rich in nutrients. This plant is commonly referred to as *Moringa oleifera* but is also known by other synonyms such as the “miracle tree” due to its numerous bioactive compounds that attract attention in traditional medicine/nutritional circles [3]. Deciduous trees or shrubs can grow up to approximately 12 m with rapid growth and the ability to survive drought. Among the various species are *M. arborea*, *M. arivae*, *M. concanensis*, *M. drouhardii*, *M. ruspolian*, *M. longituba*, *M. stenoprtala*, *M. peregrina*, *M. pygmaea*, *M. borziana*, *M. hildebrandtii*, and *M. ovalifolia*. These plants have medicinal value owing to the presence of natural compounds that can be used for treatment. In this area, one tree known as *Moringa oleifera* stands out because it has a variety of health-related positives. Normally growing between 10 and 12 m tall, *Moringa oleifera* is a small but fast-growing evergreen and deciduous tree. Leaves, which are tripinnate in nature, have a spreading crown characterized by delicate drooping branches and dense corky whitish bark [4].

All parts of the plant contain proteins, vitamins, minerals, and carotenoids, which make up the overall nutritional profile worldwide. Poor nutrition among infants and lactating mothers has been addressed with the use of *Moringa* trees [5]. Essential nutrients, such as proteins and minerals (including vitamins A and C), are abundant in leaves. Additionally, *Moringa* is equipped with approximately 46 antioxidants; hence, it is one of the most potent natural sources of these substances. Antioxidants play a key role in destroying the negative effects of free radicals on the human body. Flavonoids, tannins, saponins, alkaloids, phenolics, and triterpenoids are active constituents of *M. oleifera,* with antibacterial properties acting as antioxidants [6]. These plant leaves can be eaten fresh, roasted, or dried, and they retain their nutritional value for several months according to reports. Other foods are scarce during the dry season. *M. oleifera* blooms towards the end of a long dry period when it can flourish into lush foliage, which means that other food sources have disappeared or diminished greatly, meaning that this plant is a possible food crop for human use [7].

The native drugs are said to be derived from a particular plant with many different types that have been used for the treatment of various diseases for generations. Furthermore, this plant is all-purpose: the leaf bark, sap flower seed oil, or root can be used medicinally throughout [8,9]. Aiding hypertension, anxiety, and diarrhea, and being a natural diuretic are some of the conditions that give *Moringa oleifera* healing properties [10,11]. It also has an effect against dysentery and colitis [12,13]. *Moringa* leaves can be used topically to treat inflammatory problems, such as glandular swelling, headaches, and bronchitis [14]. The pods are good for the liver while relieving joint pain [15]. In herbal medicine, the roots cure kidney stones [16], liver ailments [17], inflammation [18], ulcers [19], and earache/toothache [20]. The gum taken from this plant is used by Indians to induce abortions and treat fever [21,22]. The seeds of this plant have laxative potential and are used to treat ailments of the bladder and prostate, and cancer [23]. Because the seeds alter oxidative stress and reduce inflammation, they have the potential to treat arthritis [24]. Preparations made from the leaves of the plant enhance overall population health and help nursing mothers and undernourished babies. These substances increase the synthesis of estrogen, which in turn provokes the growth of mammalian tissue, resulting in the secretion of milk. They are instrumental in addressing malnutrition in children under three years of age. The leaves are also valuable for individuals with insomnia [25] and wound care [26]. *Moringa* has gained widespread use in the cosmetic industry today. Historically, it was similarly employed in ancient Egypt to make skin ointments [27]. The capacity of *M. oleifera* plants to purify aquaculture wastewater has been evaluated. Studies have demonstrated its ability to concurrently remove water cloudiness, suspended solids, and microorganisms, potentially replacing or supplementing traditional coagulants and thus offering economic, health, and environmental benefits [28].

*Moringa* is renowned for its exceptional nutritional profile, offering a multitude of vitamins and minerals that far exceed those found in many common foods. It boasts seven times the ascorbic acid (vit C) found in oranges, 10 times the retinol (vit A) of carrots, 17 times the calcium of milk, nine times the protein of yogurt, 15 times the potassium of bananas, and 25 times the iron of spinach [29]. This plant is a powerhouse for essential minerals, with calcium being a standout for human development. For instance, while eight ounces of milk provide 300 to 400 mg of calcium (an important element of bone density), this plant’s leaves offer 1000 mg, and *Moringa* powder can deliver over 4000 mg of bone density. Anemia can be treated using *Moringa* powder as an alternative to iron pills. The iron content in beef is only 2 mg, whereas powdered *Moringa* leaves contain 28 mg. One of the essential elements of zinc is obtained through nutrition for sperm cell synthesis and growth and for nucleic acid synthesis. The amount of this element found in *M. oleifera* leaves constitutes about 25.5–31.03 mg/kg, which is the recommended everyday allowance for zinc in a healthy diet [30]. Tree bark is thought to be highly helpful in treating a variety of conditions including ulcers, toothaches, and high blood pressure. In addition, it has been discovered that the roots can help treat paralysis, helminthiasis, and toothaches. The flowers are used to produce aphrodisiac compounds, cure ulcers, and enlarge the spleen [14,31,32]. Temperatures between 25 and 35 °C are ideal for growing *M. oleifera* in all semi-tropical and tropical parts of the planet. It requires loamy or sandy soil with a pH range of mildly acidic to moderately alkaline and 250–3000 mm net precipitation [33,34]. *M. oleifera* differs in the distribution of nutrients at various sites. The nutritional components of plants cultivated in Nigeria and India differ slightly [35]. Recent pharmacological research has uncovered a variety of therapeutic properties of different extracts of *Moringa*, including antimicrobial, antifungal, anti-inflammatory, antioxidant, anticancer, fertility-enhancing, and wound-healing effects. This review aims to consolidate the latest findings on the pharmacological actions, global research, toxicology, phytochemistry, and overall attributes of *M. oleifera*.

### Traditional Uses of Moringa oleifera in Herbal Medicine

**Figure 1:**
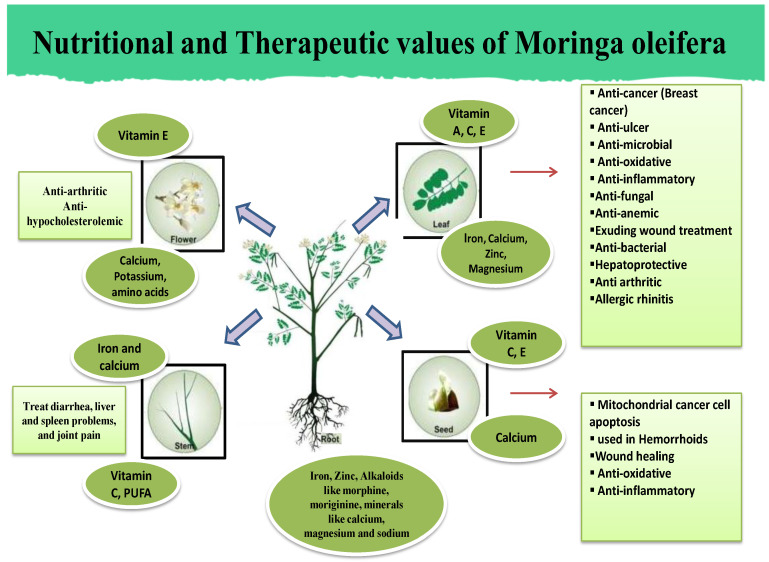
Nutritional and therapeutic values of *Moringa oleifera* whole plant.

In accordance with the tenets of Traditional Ayurvedic Medicine, the physiological characteristics and therapeutic utility of various components of *Moringa oleifera* are projected to elicit a diverse array of effects contingent on the individual’s bodily constitution and their alignment with Vata or Kapha doshas. *Moringa oleifera* manifests bitterness and acridity, is imbued with thermogenic properties, fosters digestion, functions as a carminative and anthelmintic as well as a constipating agent, mitigates pain, and exhibits anti-inflammatory effects. Moreover, it facilitates menstruation, induces sweating, enhances urine production, promotes ocular health, engenders local warmth, aids expectoration, elevates hemoglobin levels, dissolves urinary stones, serves as an antidote, stimulates physiological functions, and evokes blistering. It rectifies imbalances associated with the Vata and Kapha constitutions, including dyspepsia, anorexia, helminthiasis, diarrhea, colic, bloating, otalgia, paralysis, inflammation, menstrual irregularities, dysmenorrhea, fever, dysuria, urolithiasis, ascites, ophthalmic ailments, cough, bronchial asthma, cardiac disorders, abscesses, and otolaryngological infections. It possesses acrid and bitter flavors, is endowed with thermogenic properties, exerts abortifacient effects, combats fungal infections, and stimulates cardiovascular and circulatory functions. Mitigating Vata–Kapha imbalances and dermatophyte afflictions is recommended to address ascites [36,37]. The nutritional and therapeutic values of *Moringa oleifera* are shown in Figure 1.

*Moringa oleifera* is distinguished by its bitter taste and cooling potency, exhibiting anti-inflammatory and analgesic effects, acting as anthelmintics, promoting ocular health, and serving as rich reservoirs of vitamins A and C. It is valuable in the management of scurvy, Vata–Kapha imbalances, wounds, tumors, inflammations, and helminthic infestations. It possesses acrid and bitter tastes, imparting a cooling sensation, exerting analgesic and anti-inflammatory effects, functioning as a purgative, reducing fever, and enhancing ocular health. It is recommended for assuaging neuralgic pain, inflammation, intermittent fever, and ophthalmic disorders [24]. *Moringa oleifera*, hailing from the Himalayas, is delineated in the Chinese herbal medicine compendium, although it remains absent from classical texts of Traditional Chinese Medicine (TCM) and lacks widespread recognition as a conventional Chinese herb. Nevertheless, a variant with wingless seeds has been identified in Southern Yunnan. Imported to China from India during the 1960s, *M. oleifera* was extensively cultivated for ornamental purposes and disseminated across provinces such as Guangdong, Taiwan, and Yunnan, where it is denoted as “lamu” in Mandarin. While its nutritional significance is duly recognized, TCM principles accentuate the thermal and gustatory characteristics of herbs or foodstuffs to determine their appropriateness for individuals based on their constitutional predispositions and thermal tendencies. Predominantly ingested for its foliage, *M. oleifera* manifests both bitter and sweet flavor profiles, with a downward refrigerating impact. Given its refrigerant nature, circumspection is warranted for individuals exhibiting cold constitutions or yang deficiency, characterized by sensations of coldness, lethargy, and other manifestations associated with cold syndrome ailments [38].

## 2. Phytochemical Composition of *Moringa oleifera*

Research on *M. oleifera* and its derivatives has delved deeply into the understanding of its many facets. More than 90 distinct compounds have been identified in the genus *Moringa*, showing potential therapeutic benefits. Proteins, amino acids [39], phenolic acids [40], carotenoids [41], alkaloids [42], glucosinolates [43], flavonoids [3], sterols [44], terpenes [45], tannins and saponins [46], fatty acids [47], glycosides, and polysaccharides [48] are only a few examples of the diverse substances that are included in this classification. Examination of the chemical composition of *M. oleifera*’s many constituents has yielded a multitude of bioactive compounds, primarily secondary metabolites. Notable among them are glucosinolates; flavonoids such as kaempferol, vanillin, and quercetin; and phenolic acids such as gallic, ellagic, chlorogenic, and ferulic acids. These materials have rich nutritional, therapeutic, and anti-microbial properties. However, the amount of these compounds in *M. oleifera* isolates can vary depending on factors such as temperature, sun exposure, soil composition, and geographic location.

### 2.1. Amino Acids

*M. oleifera* contains more than 90 nutritious chemical components, such as proteins, lipids, carbohydrates, and dietary fibers. Of the several nutrients present in various sections of *M. oleifera*, proteins are the most prevalent, accounting for approximately 25% of the plant’s dry weight [49]. This plant is known to contain at least 19 distinct amino acids, including both essential and non-essential amino acids. Approximately 30% of the dry weight of seeds is composed of lipids, primarily stearic acid, saturated palmitic acid, and oleic acid [50]. The primary components of *M. oleifera* leaves are lipid molecules, palmitic acid, and linolenic acid. Furthermore, the high nutritional value of dried leaves indicates that the plant is a valuable food source [51].

### 2.2. Vitamins and Minerals

The entire *M. oleifera* plant contains several vitamins and minerals. Among the many minerals found in *Moringa* that are essential for human growth and development, calcium is thought to be one of the most vital minerals. Fresh *M. oleifera* leaves are thought to provide around 11,300 to 23,000 International Units of vitamin A. Vitamin A is necessary for many physiological activities, including vision, development, prenatal growth and development, immunologic competency, cell division, proliferation of cells and a process called apoptosis, maintenance of epidermal tissue, and cognitive function [52]. Nutritional information for *M. oleifera* is presented in Table 1.

### 2.3. Flavonoids

The wide variety of bioactive compounds found in *Moringa oleifera* seeds and leaves are highlighted in this text as having possible medical benefits. The leaves and seeds of *M. oleifera* are rich in flavonoids such as myricetin, rutin, kaempferol, quercetin, isorhamnetin, procyanidin, and catechin. In addition, there are noticeable amounts of lutein pigments in leaves [3]. The claimed therapeutic advantages of *M. oleifera* are attributed mainly to its active components. Gas chromatography–mass spectrometry examination revealed over 35 chemicals in the leaves, including β-sitosterol, pregna-7-dien-3-ol-20-one, palmitoyl chloride, cis-vaccenic acid, 5-O-acetyl-thio-octyl, tetradecanoic acid, and α-l-rhamnofuranoside [47,48,53].

### 2.4. Phenolic Acids

Sinapic, syringic, cinnamic, gentisic, and ferulic acids, along with gallic, protocatechuic, caffeic, epicatechin, p-coumaric, o-coumaric, and vanillin are found in *M. oleifera*. These compounds belong to a larger group of phenolic chemicals, obtained mainly from the naturally occurring hydroxycinnamic acid and hydroxybenzoic acid found in plants. Phenolic acids present in food have garnered increasing attention owing to their impact on individual health. These substances have been extensively studied for their beneficial effects, including anti-mutagenic, anti-inflammatory, anti-cancer, and antioxidant properties. They are abundant in fruits and vegetables and have also been present in notable amounts in the leaves of *M. oleifera*, a plant known for its nutritional value [54]. Among these phenolic acids, gallic acid was the most prevalent in dried *M. oleifera* leaves, with a mass of approximately 1.034 mg/g of dry weight. Interestingly, previous studies have reported only minimal levels of gallic acid. Additionally, chlorogenic acid and caffeic acid were found in varying concentrations, from 0.018 to 0.489 mg/g and 0.409 mg/g, respectively [44].

### 2.5. Alkaloids

Alkaloids are a group of basic nitrogenous compounds. These nitrogen atoms form amines that can be categorized as primary, secondary, and tertiary. Their therapeutic properties make them highly attractive. Alkaloids have been identified in *M. oleifera* leaves, including two newly discovered alkaloids, marumoside A and B, and aurnatiamide acetate isolated from the roots [42,46]. In addition, the stem of the plant contains moringinine and moringin-type alkaloids [47]. Glucosinolates are abundant in *M. oleifera*, and 4-O-(L-rhamnopyranosyloxy)-benzyl glucosinolate, or glucomoringin, is the most prevalent glucosinolate in the species [43]. The seeds and leaves contain the sterol isolate sitosterol, whereas the bark contains -sitosterol-3-O-D-galactopyranoside, another sterol glycoside [3,43]. Furthermore, the leaves contain an array of diterpenes and terpenes, with phytol being a notable diterpene alcohol found abundantly owing to its association with chlorophyll. Trace amounts of terpenes and their derivatives, such as farnsylacetone, linalool oxide, isolongifolene, ionone, and ionene, are present, with hexahydro-farnesyl acetone being more abundant [44].

### 2.6. Tannins, Fatty Acids, and Sterols

Natural polyphenols called tannins are found in a wide variety of plants, including fruits, vegetables, and seeds. Tannins are commonly employed in the wine industry as wine fining agents and color stabilizers, and to balance the complexity of wines by inhibiting enzymes in infested fruits. The concentration of tannins in the *Moringa* tree varies, with dried leaves having the maximum concentration (20.7 mg/g). Seeds also contain a small quantity of tannins. Out of the total antioxidant content, the proportion of total tannin content in the seeds amounts to 0.890 ± 0.020 mg GAE/g of dry matter. *M. oleifera* seeds include major fatty acids such as arachidic acid, octacosanoic acid, oleic acid, and palmitic acids. This plant also contains other fatty acids such as stearic acid, linolenic acid, behenic acid, and paullinic acid. *M. oleifera* seeds and leaves contain the sterol isolate β-sitosterol. Bark from *M. oleifera* has been used to extract β-sitosterol-3-O-β-D-galactopyranoside, another form of sterol glycoside. These tannins and sterols are responsible for various pharmacological actions [44]. Further nutritional information has been presented in Table 2.

Table 3 displays the structures of some of the major phytoconstituents separated from *M. oleifera.*

## 3. Extraction Methods and Analytical Techniques for Phytochemical Analysis

The utilization of natural substances continues to garner significant attention, owing to their numerous advantages. *Moringa oleifera* has demonstrated diverse applications in the pharmaceutical, food, cosmetic, and other sectors. This review examines the constituents of *Moringa oleifera*, such as seed extract, seed oil, and leaf extract, and details their isolation methods, processing techniques, phytochemical compositions, and modern uses. *Moringa oleifera* contains notable levels of phytochemicals, including flavonoids, carotenoids, phenolics, tannins, chlorophylls, saponins, tocopherols, and sterols [65]. The pronounced antioxidant activity of *Moringa oleifera* confers various pharmaceutical benefits across different parts of the plant, including anti-obesity, antihypertensive, anti-cancer, anti-diabetic, anti-arthritic, anti-inflammatory, and wound healing properties. Moreover, the substantial nutritional value and antimicrobial properties of the various components of *Moringa oleifera* render it suitable for diverse applications in the development of functional food products, skincare ingredients, the biodiesel industry, water treatment processes, and pharmaceuticals.

### 3.1. Extraction and Processing

#### 3.1.1. Conventional Methods

The simplest way to extract chemicals is through solid–liquid extraction, which involves transferring solutes from a solid substance into a solvent and then separating the solvent to obtain the desired extract. In one extraction method, *Moringa* leaf powder was mixed in a 70% ethanol solution for 48 h at room temperature, and the mixture was stirred every so often to aid in extraction. In contrast, others used a cold maceration method in which *Moringa* leaves were soaked for five days at ambient temperature in 70% ethanol. Another extraction process using methanol as a solvent with a solid-to-solvent ratio of 1:15 and shaking at 500 rpm for five hours at 30 °C produced a much greater yield of 14%, demonstrating the effectiveness and affordability of the maceration technique [64].

Other solvent combinations were also investigated, such as macerating *Moringa* leaves for two days at ambient temperature in an ethanol–water mixture (7:3) and extracting the leaves for 72 h in an ethanol–water solution (80:20%). After extraction, the solvent needs to be filtered and evaporated under low pressure; alternatively,, the extract can be dried in an oven or in air to produce the finished product. The percentages of extract yields observed were 14.30% for acetone, 14.50% for ethanol, 5.50% for water, and 17.50% for ethanol and water. Interestingly, clean water did not remove *Moringa* leaves as well as it could have. Therefore, a mixture of solvents might work better to make it easier to extract the various polar chemicals found in the leaves. In another method, Soxhlet extraction was performed using *n*-hexane solvent for nine hours at 70 °C and a solid-to-solvent ratio of 1:10. An effective method for extracting *Moringa* leaves with an organic solvent is the Soxhlet device. Using Soxhlet extraction apparatus with 95% ethanol over an 8-h period produced a 10.7% yield of *M. oleifera* leaf extract. Other researchers have used methanol as the solvent of choice for Soxhlet extraction and obtained a greater yield (18.56%). To achieve the final extract, the solvent is usually removed by partial vacuum drying; nevertheless, the high temperature of the extraction process may damage the delicate bioactive components [66].

#### 3.1.2. Non-Conventional Methods of Extraction

Ultrasonic-assisted extraction (UAE) is a particularly successful extraction technique that includes sonication of *Moringa* leaves with water and 0.1% formic acid at room temperature for 15 min. Conversely, 100% ethanol was used to sonicate *Moringa* leaves at a solid-to-solvent ratio of 1:10 for 30 min. Other researchers studied the effect of UAE at different temperatures (30, 35, 40, and 45 °C) and durations (15, 20, 25, and 30 min) on *Moringa* leaves with a solid-to-solvent ratio of 1:50 (g/mL). In another method, it was found that 45 °C and 30 min were the optimal conditions, as higher temperatures increased the phytochemical solubility and mass transfer rate into the solvent. UAE at 50 °C with pure water, 50% ethanol, and 20% ethanol, a constant frequency of 20 kHz, and an amplitude of 79 mm, showed that water might increase the initially determined extraction rate as well as phenolic content in *M. oleifera* leaf extract. Ultrasound waves may pass through the extraction liquid and cause cavitation, which aids in breaking the leaf cell wall and facilitates phytochemical release during extraction. Furthermore, this study focuses on the phytochemicals found in *Moringa* leaves, such as alkaloids, saponins, tannins, steroids, phenolic acids, flavonoids, glucosinolates, and anthocyanins. These chemicals provide medical benefits and can be used in the pharmaceutical, cosmetic, and food industries. The concentrations of phytonutrients in *M. oleifera* leaf extract depend on the extraction process, solvent type, solvent-to-solid ratio, ambient temperature, stirring rate, and particle size. The petroleum ether and dichloromethane samples of *M. oleifera* roots, which shown encouraging biological activity, were subjected to GC-MS (Gas chromatography and Mass Spectroscopy) analysis, which identified 102 constituents. These ingredients are classified into 13 different types of compounds, which include halo compounds, aromatics, alkamides, cyanides, fatty acids, alcohols, pyrazine, hydrocarbons, urea and *N*-hydroxyimine derivatives, unsaturated alkenamides, alkyne, and indole. A total of 39 chemicals from nine classes were found in the petroleum ether extract of the roots, according to GC/GC-MS analyses [66].

## 4. Pharmacological Activities

### 4.1. Antioxidant Properties and Mechanisms

**Figure 2:**
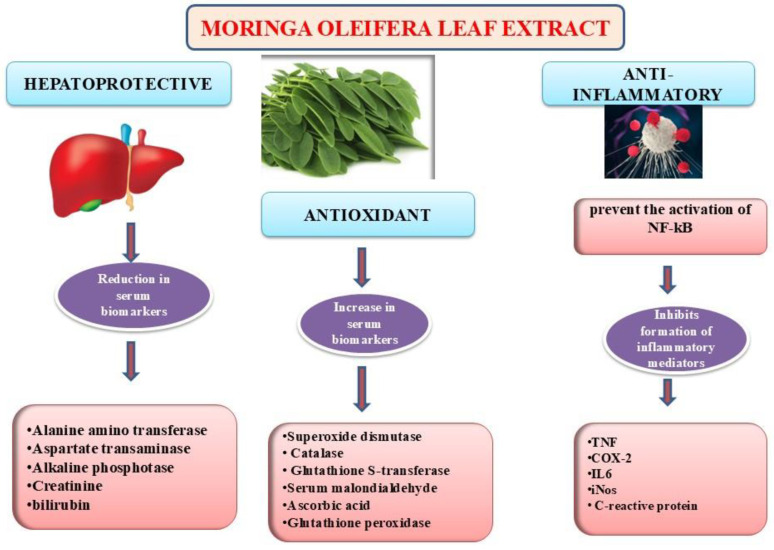
Hepatoprotective, antioxidant, and anti-inflammatory mechanisms of *M. oleifera*.

*Moringa oleifera* leaves have a powerful array of antioxidants because of the presence of a wide range of natural bioactive compounds, such as phenolics, vitamins A, E, and ascorbic acid, as well as flavonoids. The aqueous, methanolic, and ethanolic extracts produced from both leaves and roots have demonstrated exceptional in vitro antioxidant capabilities, highlighting their quantity of antioxidant components and potential for treating oxidative stress-related diseases in animal models. The administration of *Moringa oleifera* leaf extract has demonstrated a discernible capacity to ameliorate oxidative damage induced by a high-fat diet. Particularly noteworthy is the significant augmentation in the activity of pivotal antioxidant enzymes, such as superoxide dismutase, catalase, and glutathione S-transferase elicited by aqueous leaf extracts, concomitant with a reduction in lipid peroxidation levels observed in both normoglycemic and diabetic rodent models. Accumulating evidence suggests that the elevated phenolic and flavonoid content of the extract may shield against oxidative injury, conferring benefits to both individuals with normoglycemia and those with diabetes [4]. Moreover, an investigation encompassing 60 postmenopausal women showed that supplementation with *Moringa oleifera* leaf powder over a span of three months resulted in notable decreases in serum malondialdehyde levels, an indicator of lipid peroxidation, coupled with elevations in levels of ascorbic acid, superoxide dismutase, and glutathione peroxidase, underscoring the formidable antioxidant capacity of this botanical specimen [67]. Figure 2 shows hepatoprotective, antioxidant and anti-inflammatory mechanisms of *M. olifera.*

### 4.2. Anti-Inflammatory Effects

The anti-inflammatory potency of *Moringa oleifera* has been substantiated by the application of extracts from diverse plant components, including roots, stems, leaves, flowers, pods, and seeds. In a mouse study, the root extract of *M. oleifera* significantly decreased the progression of paw edema, similar to phenylbutazone, a nonsteroidal anti-inflammatory medication known for its analgesic and antipyretic properties [68]. In addition, acetylcholine-induced bronchospasms were diminished and airway inflammation in guinea pigs was alleviated by Th1/Th2 cytokine modulation using *Moringa oleifera* seed butanol extract. In addition, a clinical trial involving subjects with mild to moderate asthma demonstrated a significant increase in Forced Vital Capacity (FVC), forced expiratory volume (FEV), and peak expiratory flow rate (PEFR) following the consumption of *Moringa oleifera* dry seed powder without any adverse effects [69]. The anti-inflammatory nature of *Moringa oleifera* is attributed to the abundance of its bioactive compounds, such as quercetin. Quercetin has an astonishing capability to inhibit NF-kB activity, which is important for the swelling process. Additionally, the greater quantities of flavonoids and phenolic acids found in *M. oleifera* leaves enhance their anti-inflammatory properties. In addition, *M. oleifera* leaf extract and quercetin have been shown to control some key signs of inflammation, including iNOS, IFN-gamma, and C-reactive protein, while reducing TNF-α and IL-6 secretion in mouse models [70]. Likewise, macromolecules such as IL-1β, iNOS, TNF-α, and NO are tremendously reduced by isothiocyanates generated from *M. oleifera* leaves, thereby displaying a profound inhibitory effect on the production of pro-inflammatory substances by RAW macrophages, apart from their anti-inflammatory properties [71].

### 4.3. Antimicrobial and Antiviral Activities

*N*-benzylethylthioformate, the aglycone derivative of deoxyniazimincin, is a bioactive molecule found in the alcoholic root extract of *Moringa oleifera*. It exhibits broad-spectrum antimicrobial and antifungal activities against numerous microbes and fungi. This potential to limit microbial development and impede fungal proliferation makes it a viable option for treating infectious diseases and fighting microbial resistance [72]. Additionally, methanolic extracts from *Moringa oleifera* leaves have been found to be effective in treating urinary tract infections caused by many different types of bacteria, including gram-negative and gram-positive bacteria. Similarly, it also exhibits antibacterial effects against common infections caused by *Klebsiella pneumoniae*, *Staphylococcus aureus*, *Escherichia coli*, and *Staphylococcus saprophyticus* [73]. The inhibitory potential of *Moringa oleifera* extracts derived from its leaves, seeds, and stems is investigated across a spectrum of fungal strains, encompassing notable species such as *Aspergillus flavus*, *Aspergillus terreus*, *Aspergillus nidulans*, *Rhizoctonia solani*, *Aspergillus niger*, *Aspergillus oryzae*, *Fusarium solani*, *Penicillium sclerotigenum*, *Cladosporium cladosporioides*, *Trichophyton mentagrophytes*, *various Penicillium* species, and *Pullarium* species. The aim of this study was to elucidate the efficacy of *M. oleifera* isolates as prospective antifungal agents against a wide array of pathogenic fungi, thus enabling the design of novel preventive strategies against fungal diseases [72]. The anti-bacterial potential of *Moringa oleifera* seeds is recognized to be associated with its special bioactive constituents, including 4-(α-l-rhamnosyloxy) benzyl isothiocyanates. Additionally, juice from the leaves of *Moringa* has an excellent anti-pathogenic capacity against microbial organisms that negatively affect human lives. Surprisingly, the result achieved by methanolic leaf extract completely suppressed (99%) *Botrytis cinerea*, a necrotrophic disease that mostly affects plants [73].

Alkaloids, flavonoids, and steroids are the only bioactive substances found in *Moringa oleifera* fruits. Because of the special makeup of their steroid rings, these components impede the growth of *Candida albicans* cells through mechanisms such as protein denaturation and spore germination suppression. This intricate interaction of phytochemicals highlights *M. oleifera*’s viability as a natural treatment for Candida infections and highlights the diverse nature of its antifungal qualities [74]. *Moringa* seed kernel extract is particularly effective against different types of *Aspergillus* and Mucor, as well as *Bacillus cereus* and *Staphylococcus aureus*. However, it seems to be less effective against *Pseudomonas aeruginosa* and *Escherichia coli*, suggesting that it could be useful in treating infections caused by these bacterial strains. Recent analysis highlights the fact that only the apolar extract derived from *Moringa oleifera* seeds exhibits antibacterial activity that is selectively directed towards gram-positive bacterial strains [75].

### 4.4. Anticancer Potential

*Moringa* plant foliage has promising anticancer properties. Through advanced scientific analysis, the compound O-Ethyl-4-(α-L-rhamnosyloxy) benzyl carbamate, in synergy with 4-(α-L-rhamnosyloxy)-benzyl isothiocyanate, niazimicin, and 3-O-(6′-O-oleoyl-β-D-glucopyranosyl)-sitosterol, was subjected to rigorous assessment of their potential as antitumor agents using an in vitro assay. The extracts were made employing different solvents like chloroform, *n*-butanol, dichloromethane, and hexane by distilling the crude 80% methanol extract of the tree’s leaves. The CellTiter 96^®^ Aqueous One Solution Cell Proliferation (MTS) assay was used to assess the extracts’ cytotoxic effect on MCF7 cells. The apoptosis investigation was carried out with Annexin V-FITC analysis and validated by Western blotting with specific proteins, namely caspase 8, p53, Bax, and cytochrome c. The outcomes demonstrated that MCF7 cells (5 μg/mL) were specifically cytotoxic to the dichloromethane extract, but MCF 10A (non-cancerous breast) cells were not considerably inhibited. Out of all the studied extracts, it had the highest possible selectivity index (SI) value, at 9.5. Additionally, it caused early apoptosis and elevated the expression of caspase 8, p53, and the pro-apoptotic proteins Bax in MCF7 cells. Apoptosis induction was found to be the action mechanism through which the active principles of this plant are likely to be responsible for anticancer activity.

**Figure 3:**
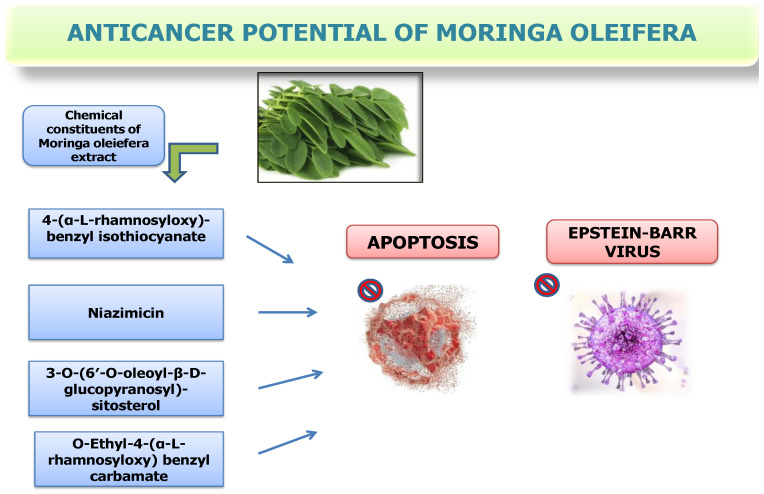
Anticancer potential of *Moringa oleifera*.

Remarkably, they demonstrated significant inhibition of the Epstein–Barr virus early antigen 36, indicating their capacity as potent antitumor promoters. Niazimicin is speculated to exert profound chemo preventive effects against carcinogenesis. Moreover, seed extracts have exhibited beneficial effects on various fronts, including antioxidant parameters, cutaneous papillomagenesis in murine models, and the modulation of hepatic carcinogen-metabolizing enzymes. These results highlight the diverse range of applications of *Moringa* chemicals in cancer research and treatment [76]. Mice with *Staphylococcus aureus* pyoderma were successfully treated with neomycin and an ointment derived from the seeds. It has been shown that niaziminin, a thiocarbamate present in *M. oleifera* leaves, inhibits Epstein–Barr virus activation brought on by tumor promoters. In contrast, tumor-promoter-driven Epstein–Barr virus activation was effectively repressed by the naturally found 4-[(4′-O-acetyl-α-L-rhamnosyloxy) benzyl] isothiocyanate, indicating the critical involvement of the isothiocyanate group in its action [76]. A crude ethanol extract prepared from dried seeds was administered to mice to reduce the inflammatory response caused by carrageenan in their hind paws. Moreover, various fractions of this crude ethanol concentrate, such as butanol, water, and hexane, showed anti-inflammatory characteristics. Remarkably, the portion containing ethyl acetate paradoxically increased inflammation and demonstrated toxicity in mice following oral consumption. Furthermore, the manufacture of Epstein–Barr virus-early antigen (EBV-EA) generated by 12-O-tetradecanoylphorbol-13-acetate (TPA) was successfully suppressed by the crude ethanol extract, indicating that it may be a promising agent against tumor promotion. A correlation between elevated levels of inflammatory mediators and reactive oxygen species (ROS) and many illnesses has been demonstrated. Therefore, it is crucial to specifically intervene in these pathways to manage the conditions associated with inflammation and oxidative stress. There is increasing interest in investigating alternative therapeutic options because of the inherent health concerns associated with standard anti-inflammatory medications. *M. oleifera* seeds have been shown to contain a variety of bioactive compounds, including nitriles, glycosidic glucosinolates, isothiocyanates, carbamates, and thiocarbamates. It has been demonstrated that these substances possess strong anti-inflammatory and antioxidant properties [77]. The mechanism of anticancer potential of *M. olifera* has been shown in Figure 3.

### 4.5. Immunomodulatory Effects

The plant extract prepared using methanol was found to contain strong active ingredients, including cyanogenic glycosides and isothiocyanates, which have shown strong immunostimulatory activities and thus greatly improve the host’s immunological response. A recent comprehensive analysis explains the deliberate application of numerous bioactive substances for the treatment of a variety of immune-mediated diseases, including cancers, hypertension, and diabetes, to boost the immune system of their hosts [77]. *Moringa oleifera* is an important research area for neuroprotection. It can improve cognitive abilities and serve as a neuroprotectant in addition to being an antioxidant in mice with dementia following amyloid beta peptide injection [78,79]. It has been demonstrated that using both aqueous and hydroalcoholic extracts from *Moringa oleifera* leaves dramatically reduces cerebral lipid peroxidation while concurrently raising brain activity levels of catalase and superoxide dismutase [78]. Additionally, a thorough analysis of the neuroprotective properties of an ethanolic extract derived from *Moringa oleifera* leaves showed significant stimulation of neurite outgrowth when primary hippocampal neuron cells were incubated with the extract. This facilitation was demonstrated by significant increases in dendritic and axonal branching as well as in length. Owing to *M. oleifera’s* capacity to lessen oxidative stress, our results unequivocally demonstrate the potential of the plant as a neuroprotective adjuvant [80].

### 4.6. Hepatoprotective and Nephroprotective Activities

Scientific data support the remarkable potential of *Moringa oleifera* leaves in mitigating drug-induced damage to the liver and kidneys in animal models. The hepatorenal protective potential of *M. oleifera* against a variety of pharmaceutical medications such as acetaminophen, gentamicin, pyrazinamide, rifampicin, and isoniazid has been elucidated in several studies. Its leaves are primarily responsible for its quality protection. More specifically, *M. oleifera* leaf extract was administered to treated rats, and the blood levels of key markers such as urea, creatinine, alkaline phosphatase, aspartate aminotransferase, and alanine aminotransferase were decreased consequently. Histological analyses corroborated these findings by showing that individuals treated with *M. oleifera* exhibited a significant reduction in drug-induced damage to the renal and hepatic systems. Furthermore, both aqueous and alcoholic extracts derived from *M. oleifera* roots and flowers exhibit notable hepatoprotective properties against acetaminophen-induced hepatotoxicity. This is evidenced by diminished levels of serum transaminases (alanine aminotransferase and aspartate aminotransferase), alkaline phosphatase, and bilirubin, suggesting a multifaceted protective mechanism conferred by *M. oleifera* [81,82].

### 4.7. Antifungal Activity

In a variety of fungal strains, including *Aspergillus flavus*, *A. terreus*, *A. nidulans*, *A. niger*, *A. oryzae*, *Rhizoctonia solani*, *Fusarium solani*, *Penicillium sclerotigenum*, *Trichophyton mentagrophytes*, *Cladosporium cladosporioides*, and *Penicillium* species, the inhibitory effect of extracts from leaves, seeds, and stems of *M. oleifera* has been identified [72]. The active ingredients in *M. oleifera* seeds, 4-(alpha-L-rhamanosyloxy) benzyl isothiocyanates, are thought to be the cause of their antibacterial activity [83]. Additionally, the juice from *Moringa* leaves demonstrated potential against harmful microbes to humans. *Botrytis cinerea*, a necrotrophic plant fungus, is nearly 99% inhibited by the methanolic extract of leaves [74]. Alkaloids, flavonoids, and steroids found in *M. oleifera* fruit have an inhibitory effect on *Candida albicans* culture by either weakening the protein or preventing spore germination due to the presence of a steroid ring [75]. *Moringa* seed kernel extract was found to have potent inhibitory effects on *Aspergillus* species, *Bacillus cereus*, *Staphylococcus aureus*, and *Mucor* species. However, its efficacy against *E. coli* and *P. aeruginosa* was reduced. This suggested that *Moringa* seed kernel concentrate might be used to treat infections that arise from all species but not *P. aeruginosa* and *E. coli* [84].

### 4.8. Cardioprotective Activity

Animals with myocardial infarction caused by isoproterenol had a cardioprotective effect in response to the freeze-dried alcoholic and aqueous extract of *M. oleifera*. Chronic *M. oleifera* treatment enhanced the levels of enzymes such SOD, catalase, lactase dehydrogenase, glutathione peroxidase, and creatine kinase and effectively treated the hemodynamic effects of isoproterenol. It has been demonstrated that in rats with isoproterenol-induced cardiac necrosis, butanolic extract is a significant source of antioxidants [85]. Moreover, because *N*-α-rhamnopyranosyl vincosamide is present, it was discovered to considerably lower inflammatory responses and myocardial necrosis [86]. *Moringa* leaves protected hypertensive rats from hypertension, resulting in markedly reduced cholesterol levels. The active ingredients niazirimin A, niazirimin B, and niazimincin were found to be the ones thought to oversee this activity [22].

Niazimin-A, niazicin-A, and niaziminin-B are among the compounds that are allegedly found in the *M. oleifera* plant extract. When these substances were directed towards (ACE), a crucial renin-angiotensin system enzyme, they were shown to have strong antihypertensive effects. By using protein–ligand docking to monitor this activity, researchers discovered that the compounds had a higher affinity for the angiotensin-converting enzyme than captopril and enalapril [87]. Rennin, an angiotensin enzyme, is a major contributor to blood pressure regulation and the development of conditions like hypertension, renal disease, and other cardiovascular disorders. In comparison to typical medications (captopril and enalapril), the study discovered that *M. oleifera*, along with two other plants (*Azadirachta indica* and *Hibiscus sabdariffa*), inhibited the enzyme with percentage inhibitions (71.8%, 74%, and 73.4%). The substance identified as β-sitosterol is what gives *Moringa* its action [88].

### 4.9. Hypocholesterolaemic and Hypolipidemic Activities

Indians employ the leaves of *M. oleifera* as a hypocholesterolemic drug in their herbal treatment for patients who are obese. Thus, the scientific rationale behind their application in hypercholesterolemia was investigated. The study revealed that the consumption of *Moringa oleifera*’s crude leaf extract in conjunction with a high-fat diet reduced the elevations in serum, liver, and kidney cholesterol levels caused by the diet by 14.35% (115–103.2 mg/100 mL of serum), 6.40% (9.4–8.8 mg/g wet weight), and 11.09% (1.09–0.97 mg/g wet weight), in that order. The impact on the blood cholesterol was statistically noteworthy. There was no discernible change in serum total protein levels. On the other hand, serum albumin rose by 15.22% (46–53 g/L) due to the crude extract. It was also discovered that this value was statistically significant. The study concluded that *Moringa oleifera* leaves possess a distinct hypocholesterolemic effect and that there is a sound pharmacological foundation for its use in India. It is also effective in reducing serum concentrations of VLDL (called very-low-density lipoprotein) and LDL (called low-density lipoprotein) by consuming fruit from *M. oleifera*. In addition to these advantages, *M. oleifera* leaf extract is also known to reduce the progression of atherosclerotic plaques. Although few human trials have been done, some research has indicated that *M. oleifera* may be helpful in treating dyslipidemia and diabetes [17].

## 5. Health Benefits and Therapeutic Applications

### Modern Therapeutic Applications Based on Scientific Evidence

The definitive findings of this investigation underscore the promising efficacy of traditional methodology in evaluating the efficiency of formulating cosmetic and natural health products. The detrimental effects of neem oil, deployed in aquaculture as an insecticide to counteract the risks posed by predators and parasites to juvenile fish, were proficiently counteracted by the application of *Moringa oleifera* leaf extract. According to the researcher’s concluding observations, *Moringa* emerges as a noteworthy dietary adjunct owing to its abundant reservoir of proteins, lipids, and amino acids, notably sulphur, alongside its minimal concentration of deleterious constituents [89]. Palmitic acid, an active constituent derived from *Moringa* leaf extract, shows a multifaceted spectrum of therapeutic attributes. A collaborative endeavor by a team of researchers delved into its pharmacological prowess, subjecting it to rigorous evaluation against an array of microbial and fungal strains. As illustrated by the accomplishment of the largest inhibitory zone among its fungal and microbial rivals, the results showed extraordinary efficiency. Extending the boundaries of innovation, *Moringa* extract was seamlessly blended into nanoparticle technology, acting as a novel drug delivery vehicle. This strategic integration not only improves drug bioavailability, but also supports a shift towards precision medicine. The use of *Moringa* is not confined only to pharmaceuticals; it is also widely used in other sectors such as poultry farming. Its huge armory includes protection from viruses such as Newcastle Disease Virus (NCDV), bacteria, and parasites, all contributing to keeping animals on earth from becoming extinct [24].

Additionally, *Moringa* serves as a noteworthy instance of an agricultural breakthrough by controlling the growth of crops in their initial stages, such as maize, tomatoes, peanuts, and cereals. This relationship promotes resilience and vigor, which enhances production volume and agricultural viability. It is also a pioneer in sustainable pest management, among other uses in agriculture. The use of naturally occurring bioactive compounds from *Moringa* to produce biopesticides not only provides a cheaper alternative but is also a shift towards sustainable agriculture. This concept democratizes pest control practices while increasing accessibility and improving resilience to different ecological settings through reduced environmental footprint [90]. Various nutrients and growth promoters, including gibberellins, cytokinins, and indoleacetic acid, are present in the aqueous extract of *Moringa oleifera*. It then becomes an effective plant bio-stimulant, convincingly safer, and thus better than conventional chemical fertilizers and pesticides used in modern farming techniques. Zeatin is an important plant hormone responsible for high drought tolerance in plants; it plays a crucial role in the ability of plants to withstand dry conditions. Consequently, compared to plants produced with proper watering, the growth properties of plants infused with methanol-based extracts of *Moringa* are greatly enhanced when they are cultivated under dry conditions. *Moringa* is used by many African tribes because it has a very low cost and can be used to enhance water quality. This can help in dealing with issues such as turbidity, alkalinity, and dissolved organic carbon. Alternatively, turbidity within water could also be reduced using *Moringa* instead of alum, indicating that it can be applied in eco-friendly water treatment techniques [4]. This makes it a better option for biopesticide applications, because it is resistant to plant diseases. *Moringa* leaves mixed with soil were found to facilitate the recovery of several plants from *Pythium debaryanum*-induced damping-off disease [1]. Incredibly, *Moringa* is said to have fruit, flowers, leaves, seeds, and roots that are useful for mental health and spiritual well-being. A range of these plants may be helpful in developing holistic healing methods that agree with both traditional knowledge and modern scientific findings [24].

## 6. Safety and Toxicity Considerations

The inquiry was a complete one that combined various experimental approaches to explore the safety limits of *Moringa oleifera*, a plant widely known for its nutritional and medicinal use. A multidisciplinary approach involved a detailed investigation of different parts of the plant with animals through leaves, seeds, and stem bark, with the aim of ascertaining possible toxicity. One aspect of this study used non-pregnant female albino Wistar rats that were exposed to aqueous methanol extract of *M. oleifera*. Following the oral administration of the extract at a dose of 2000 mg/kg to a randomly selected cohort, blood samples were obtained for analysis. Aspartate aminotransferase (AST), alanine aminotransferase (ALT), and total bilirubin levels were important parameters that were closely monitored. From the experiments, it was noted that the lethal dose in female Wistar rats exceeded 2000 mg/kg by aqueous extract, indicating a good safety rating for the administered dose [91]. The aim of this study was to determine the safety of *Moringa* leaf powder in Sprague-Dawley rats. The research was very broad to check for possible dangers or death caused by oral administration of sun-dried leaves at various doses of up to 2000 mg/kg. Its safety has been confirmed in several extensive tests, which clearly showed that no adverse effects occurred and therefore renders *Moringa* leaf extract safe within the stipulated dosage limit. The toxicological profile of *M. oleifera* seeds is an important field that needs much scrutiny. Methanolic extracts were used by scholars in their study, which involved acute and sub-acute toxicity assessments in animals aimed at establishing their health effects. Symptoms of acute poisoning were observed when the threshold dose reached 4000 mg/kg. Unfortunately, deaths have been recorded at high doses, especially above 5000 mg/kg, which is also known as the fatal dose. No negative effects were noted at values lower than 3000 mg/kg. When the seed extract was administered sub-acutely, the weight of the experimental rats decreased significantly (*p* < 0.05) and the concentrations of ALT and AST (alanine and aspartate transferases) increased significantly (*p* < 0.05) at a dose of 1600 mg/kg. This analysis concludes that *M. oleifera* seed extract is safe for use in both nutrition and medicine [92]. Although short-term harmful effects have been observed, studies have shown promising prospects for utilizing *Moringa oleifera* seed extract for nutritional purposes. The acute manifestations observed in this study underscore the need for careful dosage considerations and more refined formulation strategies to reduce the risk of adverse events.

Nonetheless, despite this immediate impact, seed extract has great potential for use in functional foods and dietary supplements that need to be investigated further, as far as these nutritional advantages are concerned [92]. Furthermore, *Moringa oleifera* stem bark extract was evaluated through oral gavage dosing up to 2000 mg/kg/day, followed by acute and sub-acute toxicity studies, which were found to be non-toxic. These findings confirm the safety of *M. oleifera* stem bark upon oral intake and its potential for medicinal use with possible bioactive components. Additionally, a subacute toxicity trial conducted over 60 days provided informative data regarding the safety profile of the extract. There were also no noticeable changes in sperm parameters (quality), biochemical, or hematological parameters (control group), as shown by the lethal dose of 1585 mg/kg after various doses ranging between 250–1500 mg/kg administered. These findings emphasize the necessity for more thorough toxicity evaluations to fully understand the safety profile of plant extracts [93].

## 7. Future Perspectives and Research Directions

Bioactive compounds obtained from the leaves, roots, seeds, and oils of *Moringa oleifera* have the potential to provide a wide range of health benefits including strong antibacterial, anticancer, antiproliferative, antihypertensive, and anti-inflammatory effects. This potential has been carefully verified by extensive studies carried out in living organisms (in vivo) and controlled laboratory environments (in vitro). The bioactive compounds derived from *M. oleifera* Lam. into the human diet is a critical step in improving nutritional profiles, promoting human welfare, and advancing sustainability initiatives in the food production sector. The intrinsic bioactivity and stability of bioactive ingredients are two key factors that determine their efficacy. These characteristics are closely related to their molecular makeup and ability to retain their bioactive characteristics when incorporated into food matrices. Optimizing the utilization of these bioactive substances in food and beverage formulations can be greatly enhanced by combining traditional and cutting-edge approaches in the chemical, thermal, and biotechnological domains. In the food industry, encapsulation is emerging as a highly promising method that provides a sophisticated way to transport bioactive chemicals from *M. oleifera* into the gastrointestinal tract while guaranteeing the sensory appeal, safety, and affordability of the end products. Therefore, in contrast to typical food offerings, developing healthful meals supplemented with *M. oleifera* bioactive compounds requires careful tailoring while keeping an eye on environmental sustainability and cost-effectiveness. Spray drying is a versatile and affordable encapsulation technique, especially when utilizing specific biopolymers, such as proteins or their derivatives, as demonstrated by a thorough literature review. The long-term durability of the enclosed materials reflects the effectiveness of the process. Future studies should be conducted to assess the environmental sustainability concerns associated with different encapsulation processes, such as the acquisition of encapsulating matrices. Despite its potential to address socioeconomic challenges, such as poverty and malnutrition, several barriers hinder the large-scale cultivation of *Moringa* trees, particularly in regions like Nepal, due to ignorance about its importance. Two other issues are the lack of awareness about farming methods, product use, and volatile market conditions. If farmers in rural areas are encouraged to grow *Moringa* trees, nutrition and health outcomes might improve, rural communities may become empowered, and more generalized socio-economic development goals can be achieved. This could generate funding for rural development activities by reducing the dependence on imported artificial supplements and medications for locally produced nutrient-dense substances.

The benefit of extracts from the *Moringa* plant is that, at the quantities and volumes often used for medicinal efficacy, they seem to be extremely safe. In the field of research, *M. oleifera* has gained widespread acceptance, and scientists have developed a variety of formulations using a variety of techniques. Table 4 lists the many phytoformulations made with *M. oleifera*.

## 8. Conclusions

Several experiments have demonstrated the myriad undeniable healing and therapeutic properties of *Moringa oleifera*. This study aimed to examine its nutritional composition and how well it can combat microbial infections, inflammation, and fibrosis. The components and mechanisms of action of plants can be researched on a large scale to bring about drastic changes in medicine. *Moringa* has medicinal value as well as being pharmacological. It is also rich in bioactive compounds, in addition to providing basic nutrition that provides additional health benefits. This synthesis provides comprehensive information about *Moringa oleifera*, considering its global exploration, ethnopharmacological findings, drug efficacy profiles, phytochemical compositions, formulation as a phytopharmaceutical, clinical validations, and toxicological profiles. As for *Moringa oleifera*, it is a potent botanical antibacterial agent owing to many biomolecules including flavonoids, tannins, saponins, alkaloids, phenolics, and triterpenoids among others. The protein and mineral contents of *Moringa oleifera* leaf extract have traditionally helped manage soft tissue infections in the mouth. In addition, it might be possible to isolate the endophytic fungus further for the identification of specific enzymes or proteins that are responsible for anti- cancer and anti-diabetic activities of *M. oleifera*, leading to the discovery of new therapeutic modalities. Therefore, by considering the economic viability of *M. oleifera* as a bio-coagulant for water treatment, examining whether it is accessible would also be a good idea. Additionally, snacks enriched with *Moringa* can be both profitable and helpful for hunger alleviation because they are in high demand by members of the population looking to have nutritious diets. If industries and researchers investigate its nutritional benefits through long-term studies for proper affirmation after earlier claims, this Indian native plant (*Moringa*) possesses huge economic potential.

## Figures and Tables

**Table 1:** Phytoconstituents of *M. oleifera* leaves per 1 g net quantity.

Phytoconstituents	Concentration	Category	Reference
Isoquercetin	1575.28 g/g (*w*/*w*)	Flavonoids	[51,52]
Astragalin	0.153 g/g (*w*/*w*)	Flavonoids	
Isorhamnetin	2.9 mg/g (*w*/*w*)	Flavonoids	
4-(_-L-rhamnopyranosyloxy) benzyl glucosinolate	33.9 mg/g	Glucosinolates	
4-[(20-O-acetyl-_-L-rhamnosyloxy) benzyl] Glucosinolate	21.84 to 59.4 mg/g	Glucosinolates	
Epicatechin	5.68 mg/g	Flavonoid	
Ferulic acid	0.078 mg/g	Phenolic acid	
Caffeic acid	0.409 mg/g	Phenolic acid	
Ellagic acid	0.018 mg/g	Phenolic acid	
Sinalbin	2.36 mg/g	Glucosinolates	
Chlorogenic acid	0.018 mg/g	Phenolic acid	
Gallic acid	1.034 mg/g	Phenolic acid	
Salicylic acid	0.14 µg/g	Phenolic acid	
4-[(40-O-Acetyl-_-Lrhamnosyloxy) benzyl]	2.16 to 5.0 mg/g	Glucosinolates	

**Table 2:** Nutritional information for *Moringa oleifera*.

Category	Subcategory	Details	Reference
Comparison of Nutrient Content	Milk	Provides 300–400 mg of certain nutrients	[48]
	*Moringa* Leaves	Offers significantly higher amounts, around 1000 mg	[55]
	*Moringa* (whole plant) Powder	Surpasses with more than 4000 mg of essential nutrients	[49]
Anemia Treatment		*Moringa* powder serves as a substitute for iron tablets, can be utilized in the treatment of anemia	[48]
Micronutrient Composition of *M. oleifera* (mg/100 g)	Calcium	440–3650 mg	[15]
	Magnesium	24–1050 mg	
	Sulfur	137–925 mg	
	Sodium	164.0–272 mg	
	Potassium	259–2061 mg	
	Phosphorus	70–300 mg	
	Iron	0.85–126 mg	
	Zinc	0.16–3.30 mg	
	Copper	0.6–1.1 mg	
Vitamin Content	Vit A	6.78–18.90 mg	
	Vit B2	0.05–20.50 mg	
	Vit B3	0.8–8.2 mg	
	Vit B7	423 mg	
	Vit B12	0.06–2.64 mg	
	Vit C	17.3–220.0 mg	
	Vit E	77 mg	

*M. oleifera* seed oil extract typically includes tannins and saponins, which oversee a variety of pharmacological actions [45]. *M. oleifera* seeds contain a variety of fatty acids, including arachidic, stearic, octacosanoic, palmitic, oleic, linolenic, behenic, and paullinic acid [46]. Two glycosides, niazirin and niazirinin, were isolated from *M. oleifera*’s ethanolic extract of *M. oleifera* [47]. Numerous polysaccharides, including D-xylose, D-galactose, L-rhamnose, L-arabinose, D-mannose, and glucuronic acid, are present in gum exudates [48].

**Table 3:** Chemical constituents of *Moringa oleifera*.

Plant Part	Phytoconstituent	Type	Chemical Structure	Therapeutic Potential	Reference
Leaves	Rutin (555.6 µg/g)	Flavonoid	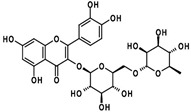	Maximum affinity towards BRAC-1 genes.	[52,56]
	Quercetin (2030.9 µmol/100 g)	Flavonoid	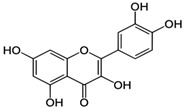	Anti-diabetic agent	[57]
	Kaempherol (197.6 µg/g)	Flavonoid	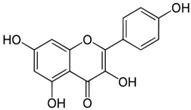	Oxidative damage protective activity.	[58]
	Isorhamnetin (0.118 mg/g)	Flavonoid	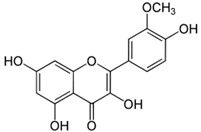	Antioxidant	[59]
	O coumaric acid (0.536 mg/g)	Phenolic acid	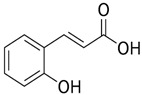	Antioxidant and antimicrobial	[60]
	Syringic acid (trace amount)	Phenol	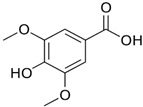	Antioxidant and antimicrobial	[60]
	Gallic acid (1.034 mg/g)	Phenol	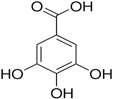	Anti-inflammatory, anti-neoplastic, antioxidant	[60]
	Sinapic acid (trace amount)	Phenol	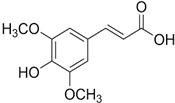	Cardioprotective, renoprotective, neuroprotective, anxiolytic	[60]
	Caffeic acid (0.409 mg/g)	Phenol	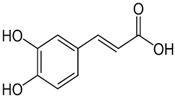	enhances athletic performance, reduces fatigue, helps weight management, protects against HIV, herpes, and cancer.	[60]
	Ellagic acid (0.078 to 0.128 mg/g)	Polyphenol	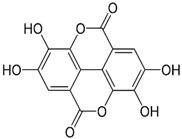	Antiviral, antibacterial and antioxidant	[60]
Leaves and Seeds	Myricetin (5.804 mg/g)	Flavonoid	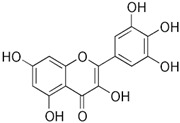	Prevention of diabetes and its complications.	[60]
Seeds	Arachidic acid	Fatty acid	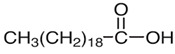	Increases mothers’ milk production	[61]
	Oleic acid	Fatty acid	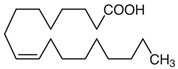	Reduces blood pressure, antioxidant	[62]
	Myristic acid	Fatty acid	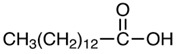	Anxiolytic	[63]
	Palmitic acid	Fatty acid	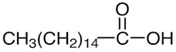	Anti-leukemic effect	[64]

**Table 4:** Different Phytoformulations of *M. oleifera*.

Part of the Plant	Formulation	Application	Reference
Leaves	Polyherbal formulation	Anti-ulcer	[94]
	Polyherbal ointments	Edema	
	Lozenges	Anti-microbial activity	
	Film dressing	Wound healing	
	Effervescent tablets	Anti-anemia	
	Granules	Anti-inflammatory and anti-arthritic	
Seed	Micro-dispersion	Anti-inflammatory	
	Nano-micelle	Mitochondrial cancer cell apoptosis	
	Anti-inflammatory cream	Anti-inflammatory	

## Data Availability

No new data were created or analyzed in this study.
